# A Remarkable Case of Inversion of the Uterus, Reduced after Twenty-two Years’ Duration

**Published:** 1872-08

**Authors:** James P. White


BUFFALO

### Medical and Surgical Journal.

VOL XII. AUGUST, 1872. No. 1.

### Original Communications.

:o:

ART. I.—*A Remarkable Case of Inversion of the Uterus. Reduced after twenty-two years Duration.* By Prof. JAMES P. WHITE, M. D.

Reported by A. T. LIVINGSTON.

The reduction of an inverted uterus, except immediately after the accident, is one of the most recent results of Obstetric Surgery.

Not merely the propriety, but even the possibility, of its accomplishment has been generally denied by Authors up to the present time.

The fallacy of this doctrine has again been beautifully demonstrated by Prof. James P. White, M. D. of this city (Buffalo), in the reduction of a complete inversion of the uterus of *twenty-two years* standing. To Prof. White is due the credit of originating this operation, which he did so long ago as 1856, when he reduced a case of eight days duration.

In connection with a report of this case published in the *Buffalo Medical Journal* for March 1856, and again, more fully, in the report of his third case, published in the American Journal of the Medical Sciences for July, 1858, Prof. White expressed the most sanguine convictions as to the feasibility of reposition in cases of long duration.

There is absolutely nothing wanting, in this latest, the tenth case operated upon by Dr. White, to entirely establish the truth of those convictions.

This case, of which we give an account below, is considered to be the most remarkable one upon record; particularly in point of *time*, which, it clearly proves, is not a condition that negatives the success of the operation.

The history of the patient prior to the time of the operation has been obtained principally from a thesis presented to the faculty of the Buffalo Medical College, in February, 1871, by Dr. O. C. Strong of Colden, N. Y.

Mrs. Aime Dubois, then aged twenty-four, and residing in Buffalo, gave birth to a female child, July 15th, 1850. The midwife, who attended her in her confinement, experienced some difficulty in removing the placenta, but finally succeeded in *pulling it away*. This act was followed by so great hemorrhage as nearly to destroy the woman's life. From this time until the day of the operation she suffered a continual loss of blood, which, twice a month. amounted to a "flooding." She consulted various physicians in regard to her condition but received no benefit from any of them This is not surprising when we are told that, until Dr. Strong was called to see her in 1870, no physician had ever proposed to make an examination per vaginam with the view to determine the cause of the hemorrhage.

March 2d, 1870, Dr. Strong was called to attend Mrs. Dubois, He "found her lying upon a bed, about which were evidences in abundance of the terrible flooding that had just recurred."

Upon making a digital examination, he discovered "a tumor about the size and shape of a small pear occupying the vagina," which, after a careful specular examination, he "diagnosed as an inverted uterus."

For this prompt and, as the sequel has shown, accurate diagnosis, Dr. Strong deserves much credit. He informed his patient that he considered her condition susceptible only of palliative measures and advised the use of tonics and local astringents. Afterwards learning of Dr. White's previous successful operations for chronic inversion of the uterus, a correspondence arose between himself and the latter with reference to the case of Mrs. D., which resulted in an invitation to Dr. White to visit the patient and, if upon examination he should think it advisable, to attempt the reposition of her uterus.

June 23d, 1872. Prof. White, with Profs. Julius F. Miner and M. G. Potter, Drs. Geo. N. Burwell and W. C. Phelps and the writer, whom he kindly invited to accompany him, proceeded to the residence of the patient in the town of Colden, Erie Co., N. Y. She was found to be feeble and very anæmic, and slight hemorrhage from the tumor was then occurring. Prof. Potter, who was requested to take charge of the anæsthetic, administered some chloroform to the patient and the tumor was then examined by several of the gentlemen present. It resembled in size and shape an ordinary hen's egg, and was suspended in the vagina by a long, narrow pedicle continuous with its smaller extremity.

This appearance, particularly the small and elongated cervix, led some who examined it to doubt its being a uterus at all and to consider it, rather, a polypoid growth. But a probe could not be passed along the pedicle into the os, as might have been done had the tumor been a polypus.

The uterus could not be detected by palpation over the abdomen. A probe passed up the vagina was felt both by the finger in the rectum and the hand placed over the hypogastrium; also the finger passed up the rectum came in contact with the anterior abdominal wall as felt by the other hand; all these diagnostic means proving the abscence of the uterus from the situation, which it normally occupies.

By these negative proofs Dr. White was entirely convinced that the tumor was the inverted uterus and he therefore proceeded to attempt the reposition of the same in the presence of the above named gentlemen who accompanied him and also Drs. Strong of Colden, Davis of Boston and G. H. Lappham of Aurora. Dr. Potter had produced anæsthesia by chloroform which he now exchanged for ether, with which he kept the patient anæsthetized during the operation.

Dr. White now assumed the kneeling posture in front of the patient, who had been placed upon the bed so that her hips projected a little beyond its edge, her feet resting in the laps of Drs. Miner and Phelps, who sat upon either side of Dr. White, each supporting a knee and holding a hand of the patient. He then introduced his right hand into the vagina and began manipulating the tumor. This manipulation consisted in compressing the uterus, which relieved its congestion and rendered it more pliable and in making gentle pressure in the line of the axis of the pelvis by use of the uterine repositor. After continuing this a short time the Doctor brought the tumor down to view, when a glance sufficed to assure the *doubting* of its true nature. By the pressure which had been exerted the neck had been shortened and dilated, the body and fundus reduced in size, the superior angles (now inferior) were distinctly seen, and altogether the tumor then present the normal outline of a uterus of small size.

The operator's uterine repositor* consists of a stem of wood or hard rubber, about ten inches in length, straight or curved, one extremity of which is enlarged and cup-shaped to fit the fundus uteri, the other extremity having attached to it a coil of steel spring wire, against which the breast of the operator may be placed, who, by leaning forward, may exert a constant and gentle pressure upon the uterus, and thus relieve his other hand, with which he can facilitate the repositing by manipulating the upper end of the uterus, either through the abdominal wall or by passing a finger up the rectum. This repositor was again introduced, and a pressure of eight or ten pounds exerted by it, and, at the same time, compression was made by the hand within the vagina upon the portion of the uterus protruding beyond the os, the same hand also retaining the fundus uteri and cup of the repositor in coaptation, the left hand being employed as above suggested. When, in this manner, the cervix had been made to embrace the fundus, the uterine repositor was substituted by a large rectal bougie, with which pressure was continued until the close of the operation.

At the end of an hour and fourteen minutes Dr. White was obliged to discontinue his efforts on account of the benumbed condition of his hand, caused by the pressure upon it of the narrow and unyielding vagina.

The fundus, at this time, was within the cervix and above the

* A full description of the Uterine Repositor, illustrated with plates, will be found in the American Journal of the Medical Sciences for April, 1872, and Buffalo Medical Journal for May, 1872.

os; and the Doctor considered the reduction substantially accomplished. He requested Dr. Miner to continue the manipulation, which he did, and in sixteen minutes, or just one hour and a half from the beginning of the operation, Dr. Miner enjoyed the satisfaction of announcing that the uterus, which for twenty-two years had been completely inverted, was now as completely reposited.

Two hours after the operation, when we left her, the patient was quite as comfortable as had been anticipated. An opiate was administered, and directions were given the attendants to keep her perfectly quiet for at least a fortnight.

By letter dated June 27th, Dr. Strong reports Mrs. D. doing better than he had dared to expect. She had complained somewhat of pain in the head and back, also of abdominal pain, which, however, was not severe. Slight tympanitis had existed, which the application of a stupe relieved. She was then taking sufficient morphine to relieve pain and keep her quiet; also quinine, wine, eggs, &c., in moderation.

| | | | | |
|---|---|---|---|---|
| June | 23, | 6 P. M., | Pulse | 90. |
| " | 24, | 8 A. M., | " | 75. |
| " | 25, | 9 " | " | 80. |
| " | 26, | 10 " | " | 90. |
| " | 27 | noon, | " | 90. |

July 2. Dr. Strong writes: "Our patient is doing well, and nothing has yet interfered with her progress toward recovery. Pulse to-day, 68. Treatment is the same as reported last, except quinine, which was withdrawn on account of gastric irritability."

The following letter, which is the latest received from Dr. Strong, presents a most satisfactory conclusion to the report of this truly wonderful case:

COLDEN, July 19, 1872.

MR. A. T. LIVINGSTON:

*My Dear Sir*—Your favor of the 14th inst. came duly to hand, and in answer I would say that since the operation upon Mrs. D. nothing has transpired to mar the beauty of the result. The pulse has at no time exceeded 90 per minute.

In two weeks from the day of the operation she began to sit in an easy chair, and since that time she has been more or less about her room. On the 14th instant an examination showed the uterus occupying its normal position, the intra-vaginal portion of the neck presenting the ordinary appearances of mild cervicitis. The patient is now about upon her feet some portion of her time, and everything points to a rapid and, I think, perfect cure. She has had no sanguineous discharges since the operation. Pulse is now normal and appetite fair.

I remain yours truly,

O. C. STRONG.

